# Heterologous Platelet-Rich Plasma in the Treatment of Severe Skin Damage

**DOI:** 10.3390/reports6030034

**Published:** 2023-07-21

**Authors:** Cristina Vocca, Francesco Romano, Gianmarco Marcianò, Vincenzo Cianconi, Davida Mirra, Andrea Dominijanni, Giovambattista De Sarro, Luca Gallelli

**Affiliations:** 1 Operative Unit of Pharmacology and Pharmacovigilance, “Renato Dulbecco” University Hospital, 88100 Catanzaro, Italy; cristina_vocca@live.it (C.V.); desarro@unicz.it (G.D.S.); gallelli@unicz.it (L.G.); 2 Department of Health Science, University Magna Graecia, 88100 Catanzaro, Italy; 3 Department of Primary Care, ASP 7, 88100 Catanzaro, Italy; romano2512@libero.it; 4 Dentirsty Unit, 00118 Rome, Italy; 5 Department of Environmental Biological and Pharmaceutical Sciences and Technologies, University of Campania “Luigi Vanvitelli”, 81100 Caserta, Italy; davida.mirra92@gmail.com; 6 Immunohaematology and Transfusion Medicine Unit, “Renato Dulbecco” University Hospital, 88100 Catanzaro, Italy; 7 Research Center FAS@UMG, Department of Health Science, University Magna Graecia, 88100 Catanzaro, Italy; 8 Medifarmagen Srl, University of Catanzaro and “Renato Dulbecco” University Hospital, 88100 Catanzaro, Italy

**Keywords:** soft tissue injury, fibrin membranes, concentrated growth factors, PRP

## Abstract

Accidental soft tissue injuries are a frequent injury. Platelet-rich plasma (PRP) is an interesting therapeutic option for wounds and skin damage. In this case report, we describe a 37-year-old man that presented to our ward of pain medicine for an accidental severe leg injury associated with skin and soft tissue loss, with severe pain and poor sensation. History revealed the use of recreational drugs without viral infections or systemic diseases. Wound debridement, wound dressings, systemic antibiotics (amoxicillin 1000 mg tid and azithromycin 500 mg od), and non-steroidal anti-inflammatory drugs (ibuprofen 600 mg bid) reduced pain but did not improve the skin and soft tissue. A fibrin membrane with concentrated growth factors was applied, yielding an improvement in the injury in 16 months without the need for skin grafting.

## 1. Introduction

Accidental soft tissue injury represents the most common management challenges for hand surgeons [1]. The management of skin and soft tissue damage requires several steps, depending on the specific nature of the lesion: (i) analgesia reduces pain; (ii) irrigation with Dakin’s solution or sterile isotonic solution reduces the development of infections; (iii) tissue debridement—removing the devitalized tissue reduces bacterial growth; (iv) dressing and/or surgical closure reduce the time of healing [2]. Previously, we reported a series of 87 patients with wound lesions in which topical application of platelet gel resolved clinical symptoms, reducing healing time [3,4,5,6].

Platelet-rich plasma (PRP) is an interesting therapeutic option for wounds and skin damage, because several growth factors are released by platelets: fibroblast growth factors, platelet-derived growth factor (PDGF), epidermal growth factor, insulin-like growth factor-1, transforming growth factor-β, and vascular endothelial growth factor [7]. PRP favors the action of growth factors and results in the formation of new epithelium; the stimulation of granulomatous tissue formation; the aggregation of fibroblasts, macrophages, and other cells; and collagen production [8]. Nevertheless, PRP has antibacterial activity against Escherichia coli, Staphylococcus aureus, Candida albicans, and Candida neoformans [9]. Moreover, the use of PRP was also reported in the healing of severe lichen sclerosus [10].

Fibrin sealants are a relatively recent therapeutic option in wound management. According to the TIME protocol (Tissue, Inflammation/infection, Moisture imbalance, and Epithelial edge advancement) for ulcer management, topical treatment has a crucial role in the recovery of these lesions. Fibrin seems to be an effective solution consequentially to its role in hemostasis; it acts in the healing process to promote collagen synthesis, angiogenesis, wound contraction, and reepithelization. It is mainly used as a sealant, adhesive, and hemostatic [11]. 

Recently, we documented the effect of this treatment in three patients with chronic wound ulcers [12]. In the present case, we report a case of successful application of fibrin membranes as a biologic wound dressing material for coverage of full-thickness soft tissue loss.

## 2. Case Report

**Figure 1:**
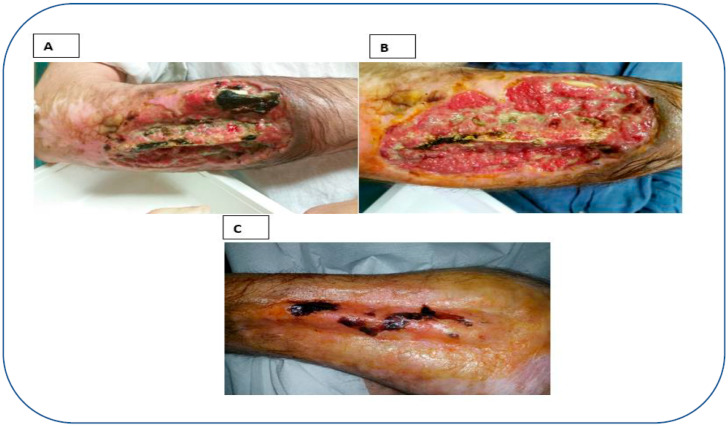
Patient’s admission conditions and management. (**A**) Skin lesion was very severe at admission, with consistent tissue damage and bone exposure. (**B**) Lesion cleansed. (**C**) Wound after treatment with platelet rich plasma + platelet poor plasma.

A 37-year-old man presented to our ward of pain medicine for an accidental severe leg injury associated with skin and soft tissue loss (Figure 1A). 

**Figure 2:**
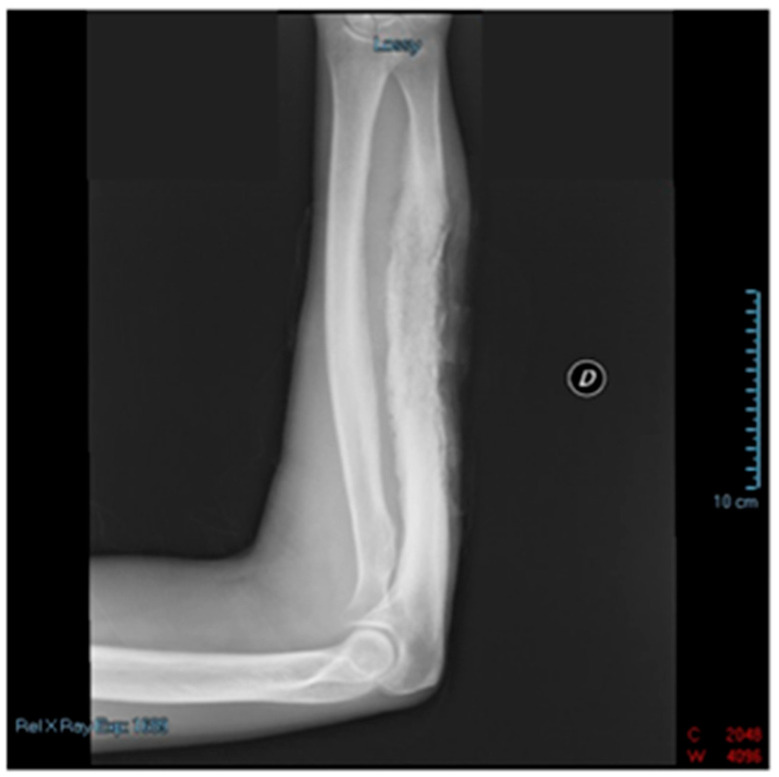
Radiograms of patient’s right arm. Despite soft tissue edema and disruption, no relevant bone involvement was documented.

History revealed the use of recreational drugs without viral infections (e.g., HIV, HBV, HCV) or systemic diseases. The patient referred that he refused the plastic surgery for tissue reconstruction and, during the home stay, he used acetaminophen and NSAIDs (ketoprofen, ketorolac, ibuprofen) for pain management without clinical improvement. At the exploration, the patient experienced severe pain (visual analogue scale, VAS 10) with poor sensation. The skin of the forearm was red with signs and symptoms of infection (fever 38.2 °C); the bone, tendons, and muscle were exposed. Interphalangeal joint flexion and extension of the last three fingers was not possible. Blood pressure was 120/78 mmHg, heart rate was 85 beats/min, and oxygen pressure saturation at room temperature was 99%. Plain radiographs excluded the presence of bone fracture but revealed a severe soft tissue edema (Figure 2). 

Initial treatment included wound debridement, wound dressings, systemic antibiotics (amoxicillin 1000 mg tid and azithromycin 500 mg od), and non-steroidal anti-inflammatory drugs (ibuprofen 600 mg bid). Seven days later, the infection of the forearm improved. The patient consented to heterologous fibrin membrane treatment, and 9 mL of heterologous blood obtained from the parents of the patient was analyzed for compatibility and sterility. About 1 week later, when the safety was shown, blood samples were taken from the parents to obtain PRP, in agreement with our previous study [12]. Briefly, blood samples were drawn in sterile Vacuette tubes (Greiner Bio-One, GmbH, Kremsmunster, Austria) without anticoagulant solutions and immediately centrifuged (Medifuge MF200, Silfradent srl, Forlì, Italy) at different velocities: 30 s of acceleration, 2 min at 872 g, 4 min at 689 g, 4 min at 872 g, 3 min at 1077 g, and 36 s of deceleration and stop. Three blood fractions resulted: (1) the upper platelet poor plasma (PPP) layer; (2) the middle fibrin-rich gel with aggregated platelets and concentrated growth factors (CGFs); (3) the lower red blood cell (RBC) layer [12].

The skin was cleaned (Figure 1B), cefepime was topically applied for 30 min, and then both fraction 2 (fibrin rich gel) and fraction 1 (platelet poor plasma) were applied. Finally, the wound was protected with an occlusive dressing.

At the follow-up (1 week later), the patient was pain-free without signs of infection (body temperature: 36.2 °C; heart rate 62 b/min). The wound was cleaned, cefepime was topically administered for 30 min, and heterologous blood was taken for membranes. The follow-up was performed every week. At one-month, no signs of infection or inflammation were present and cefepime was discontinued.

**Figure 3:**
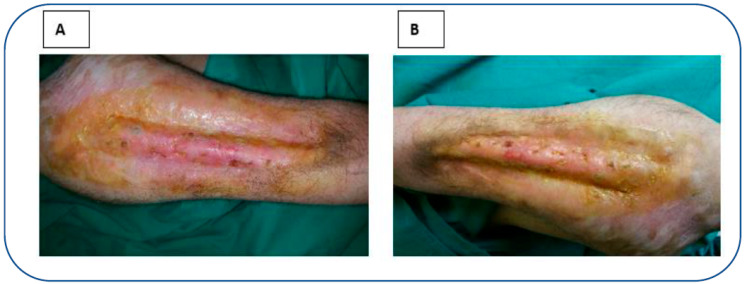
Follow-up at 4 (**A**) and 6 (**B**) months after the first admission.

During a four-month follow-up, the lesion improved (Figure 3A), and two months later (six months after the admission, Figure 3B), the patient achieved a satisfactory cosmetic appearance of the reconstructed digit with complete functional restoration. The patient recovered thermal, pain, and tactile sensitivity, evaluated using cold and hot water (thermal); a needle tip test (pain); and a cotton ball, bristle brush, and strip of paper (tactile) testing, respectively. 

## 3. Discussion

We report the clinical efficacy of fibrin membranes plus growth factors in the treatment of a traumatic non-healing ulcer. The use of topical treatment reduces surgery, offering a conservative strategy, with less stress and economic expense for patients and health care systems. Nevertheless, topical treatment is often a fundamental complement after surgery [2,11]. 

Fibrin sealants are biocompatible products. There are two main types of fibrin sealants: homologous and heterologous. The homologous type is the most diffused and validated in clinical studies, but has high costs [11]. 

Fibrin has been used in different wound management settings and in association with other components including keratinocytes, fibroblasts, and platelets [11]. Kirsner et al. [13], in a phase II clinical trial, highlighted the efficacy of a spray (0.5 × 10^6^ cells/mL every 2 weeks) containing neonatal keratinocytes, fibroblasts, and fibrin sealant in 228 venous ulcer patients. The wound area was significantly reduced in the treatment group with respect to the control one. Few adverse events (AE) were reported, including skin ulceration and cellulitis. Asadi et al. [14], in 10 patients affected by refractory ulcer, documented an improvement in symptoms after topical treatment with PRP, fibrin glue, and collagen matrix. In this study, 9 of 10 patients completely recovered, showing relevant benefits in epithelization, vascularization, and granulation tissue development, without the development of AE.

Despite these interesting results, some concerns about homologous fibrin sealants remain (e.g., high costs and risk of infection). Therefore, scientists are testing heterologous fibrin in experimental animals [11], even if, to date, only few clinical trials have been performed. 

Gatti and colleagues [15] observed a good clinical response in 13 patients with venous ulcer managed with heterologous fibrin, essential fatty acids, and Unna’s boot. The main advantages of heterologous fibrin were the decrease in pain, the ease of application, and the absence of both infection and AE. Abbade et al. [16] highlighted a clinical improvement in 10 patients managed with heterologous fibrin, a gauze soaked in fatty acids, and Unna’s boot, without the development of AE. The same group described the development of local AE related to this treatment (ulcer pain, peri-ulcer eczema, the opening of new ulcers, peri-ulcer maceration, peri-ulcer pruritus, critical colonization, and increased ulcerated area) in 31 patients [17].

A long-running debate has animated the scientific community concerning PRP use. Despite its current application (autologous and allogenic) in stimulating tissue growth and regeneration, the consensus remains controversial. Several clinical studies in non-healing ulcers demonstrated a certain grade of efficacy of this treatment [14]. 

A longitudinal single-arm trial by Mohammadi et al. [18], in 100 diabetic patients with foot ulcers, documented the efficacy of PRP gel preparation in the improvement in wound area. Suthar et al. [19] showed, in 24 patients with non-healing ulcers of different etiologies, that subcutaneous PRP followed by topical administration led to wound size reduction, without the development of autoimmunity or infections. Burgos-Alonso et al. [20], in a randomized pilot study, in 12 patients with leg wounds due to venous insufficiency showed the efficacy of autologous PRP (reduction in ulcer size by 3.9 cm^2^ for week) compared to the standard of care (time of healing 10–12 weeks). Nolan et al. [21] performed a randomized clinical trial (RCT) in 18 diabetic foot ulcer patients, divided into three arms: autologous fat grafting, autologous fat grafting plus PRP, and routine care. Although no clinical difference was shown between these groups, increased graft survival in the PRP arm was documented, increasing micro-vascularization. It is not clear if reduced apoptosis or increased proliferation are responsible for this beneficial effect. However, the increased angiogenesis related to PRP use has been highlighted as a determining factor to revert the ulcer [7]. A systematic review and meta-analysis of the existing randomized trials of PRP use in chronic wounds by Meznerics and colleagues [22] concluded that PRP is an effective method in this setting, offering great advantages. Concerning ulcer etiologies, they found a higher efficacy in venous ulcers, whereas diabetic ones had worse outcomes. This could be related to (i) the pathogenesis of diabetic ulcers or (ii) the PRP administration (commonly through injection). 

The efficacy of topical application of platelet gel in patients with skin lesions has been previously documented [3,4,5,6]. Jiritano et al. [3] and Serraino et al. [6] documented the effect of autologous PRP application in the prevention of surgical infections. Similarly, Serra et al. [5] in a diabetic patient reported the efficacy and the safety of PRP in wound healing, without the development of infections.

A low number of studies were conducted using PPP. Previously, we documented the effects of PPP + PRP coadministration in patients with wound diseases [12]. Setta and colleagues [23] recruited 24 patients with diabetic ulcers, dividing them into two groups managed, respectively, with PPP and PRP. The wound healing was significantly faster in the PRP group vs. the PPP one.

In the present case, we report the efficacy and the safety of both PPP and PRP use in a patient with traumatic non-healing ulcer that usually receives surgical treatment. Few studies were conducted on this topic in acute settings and trauma. 

The coadministration of PPP + PRP in this case is crucial because, as previously reported [12], PRP is rich in aggregated platelets and concentrated growth factors that stimulate fibroblasts, macrophages, and mesenchymal cells, while PPP contains a concentration of growth factors that can help stimulate healing and also provide extended anti-inflammatory relief and is involved in re-epithelialization and neovascularization.

It is important to underline that the presence of CGFs represents an important point for the effectiveness of PRP in the present case, as also reported by other authors in patients with severe lichen sclerosus [10]. Kazakos et al. [24], in 59 patients with acute wounds (open fractures and closed fractures with skin necrosis and friction burns), documented that the wound healing rate was significantly faster at weeks 1, 2, and 3 in patients (*n* = 27) treated with the topical application of PRP gel vs. patients (*n* = 32) treated with conventional dressings (*p* = 0.003, *p* < 0.001, and *p* < 0.001, respectively). Moreover, the authors reported that the mean time to plastic reconstruction in PRP-treated patients was 21.26 days vs. 40.6 days in dressing-treated patients. These data suggest that PRP gel treatment represents an effective aid in the treatment of acute wounds trauma. Similarly, Moneib et al. [25] documented in 40 patients with chronic venous leg ulcers that a 6-week treatment with autologous PRP (1 administration/week) induced a significant improvement in the ulcer size with respect to a 6-week treatment with compression and dressing (4.92 ± 11.94 cm and 0.13 ± 0.27 cm, respectively).

Fibrin sealants may offer a comfortable solution and effective solution. However, the cost–benefit ratio must improve, especially for the homologous preparation. Heterologous fibrin sealants are less expensive but increase the risk of immunogenicity [11]. Conversely, PRP is an optimal choice, considering its provenance from the patient and the absence of immunogenicity. Nevertheless, long-period treatment may make this treatment uncomfortable for patients, according to the necessity of blood sampling. Therefore, allogenic PRP is another possible option showing good clinical efficacy [14].

In our study, we used heterologous blood because we need a high quantity of blood for a long period, obtaining a good clinical efficacy without the development of AE or autoimmunity.

In conclusion, in the present case, we documented that the coadministration of PPP and PRP rich in CGFs represents an efficacy and safety treatment for soft tissue wounds or damages. However, larger randomized clinical trials are needed to have more solid data about its usage.

## Data Availability

All data generated during this study are included in this article. Further enquiries can be directed to the corresponding author.
